# Too much information: Why CDCL solvers need to forget learned clauses

**DOI:** 10.1371/journal.pone.0272967

**Published:** 2022-08-26

**Authors:** Tom Krüger, Jan-Hendrik Lorenz, Florian Wörz

**Affiliations:** Institute of Theoretical Computer Science, Universität Ulm, Ulm, Germany; Kingston University, UNITED KINGDOM

## Abstract

Conflict-driven clause learning (CDCL) is a remarkably successful paradigm for solving the satisfiability problem of propositional logic. Instead of a simple depth-first backtracking approach, this kind of solver learns the reason behind occurring conflicts in the form of additional clauses. However, despite the enormous success of CDCL solvers, there is still only a limited understanding of what influences the performance of these solvers in what way. Considering different measures, this paper demonstrates, quite surprisingly, that clause learning (without being able to get rid of some clauses) can not only help the solver but can oftentimes deteriorate the solution process dramatically. By conducting extensive empirical analysis, we furthermore find that the runtime distributions of CDCL solvers are multimodal. This multimodality can be seen as a reason for the deterioration phenomenon described above. Simultaneously, it also gives an indication of why clause learning *in combination with* clause deletion is virtually the de facto standard of SAT solving, in spite of this phenomenon. As a final contribution, we show that Weibull mixture distributions can accurately describe the multimodal distributions. Thus, adding new clauses to a base instance has an inherent effect of making runtimes long-tailed. This insight provides an explanation as to why the technique of forgetting clauses is useful in CDCL solvers apart from the optimization of unit propagation speed.

## 1 Introduction

Since their inception in the mid-90s [1, 2], CDCL solvers have proven enormously successful in solving the satisfiability problem of propositional logic (SAT). As a case in point, we refer to the annual SAT Competition. The goal of this competition is to promote further improvements in the field of SAT solving by hosting a competitive event where researchers can present their newest implementation work. All submitted solvers are put up against each other to solve a pool of instances, and the fastest solver wins. We refer to http://www.satcompetition.org for more detailed information. In this competition, CDCL solvers won many of the last competitions. In many combinatorial fields, applied problems are nowadays even solved by reducing the problem to a SAT instance and invoking a CDCL solver (see e. g. [3]), despite the NP-completeness of the SAT problem. SAT solvers have also been used in the last few years to generate computerized proofs of long-standing open problems. We refer to [4] for a solution of the Boolean Pythagorean Triples problem, [5] for Schur number five, or [6] for the resolution of Keller’s Conjecture with SAT solvers.

The abbreviation CDCL stands for *conflict-driven clause learning*. The eponymous component of CDCL is *clause learning*, a mechanism that can enhance the simple exhaustive exploration of the search tree for possible satisfying assignments by learning from mistakes made and avoiding these in the future. During its execution, the solver learns additional clauses containing this learned information (we refer to Section 2.2 for an introduction to CDCL solvers). This enables the solver to prune the search tree and avoid re-exploring similar parts.

In addition to a clause learning scheme, the majority of modern CDCL solvers also employ a technique to delete some of the new clauses from time to time when they were deemed not helpful by the solver (e. g., CaDiCaL and Kissat [7] even sometimes flush almost all learned clauses). However, it is still largely a mystery whether this deletion process is only used to keep computation times low by having a manageable clause database or if there is some theoretical benefit to deleting clauses.

Although both techniques, clause learning and clause deletion, are routinely employed in modern CDCL solvers, the theoretical underpinnings of the interactions between the two techniques are not entirely understood [8].

### 1.1 Our contribution

To study the effect of learned clauses on CDCL solvers, we let Glucose 4.1 (see [9, 10]), a leading CDCL solver, first learn the set L of all conflict clauses it encounters until a solution of a given instance F is found. For this, we have chosen a diverse set of satisfiable as well as unsatisfiable instances. We refer to Section 3. In a second step, we generate a multitude of different sets *L*, where each *L* is a randomly sampled subset of L. Finally, we call Glucose 4.1 on the extended instance F∪L. This model can be thought of in the following way: we simulate that the solver learns the clauses L and then aggressively deletes some of the learned clauses (only keeping the ones in *L*) and forgets its current assignment (we refer to this as a “**reset**”).

Since CDCL is nowadays the leading paradigm of successful SAT solvers, one is tempted to conjecture that clause learning is always helpful. However, using the described modification process, we demonstrate in this paper that one must be careful about this assumption. More specifically, we show that there are a surprising number of instances where the mean runtime of the extended instances is dramatically worse than the runtime on the original instance. This also holds, using runtime-independent measures like the number of conflicts or propagations that occurred towards a solution. This observation shows that the observed deterioration phenomenon cannot solely be explained by a decrease in the unit propagation speed. In particular, we have designed our sampling process in such a way that the size of the formula does not increase too much, and unit propagations can be performed at a reasonable cost.

Furthermore, the performance decrease is so substantial that it cannot be explained by pure chance. This motivates the study of the runtime distribution of extended instances to shed light on the question of what influence learned clauses have on CDCL solvers. Focusing on the runtime distribution, we obtain as our next result that the runtime distribution of Glucose 4.1 is multimodal. The observed multimodality contrasts the recently obtained result that the runtime distribution of stochastic local search (SLS) SAT solvers can be described with a single distribution (namely, a lognormal distribution) [11].

We continue our study to determine what kind of distribution type can be used to describe this multimodal data. We demonstrate that the runtimes of Glucose 4.1 are mixed Weibull distributed by conducting various statistical analyses. These distributions possess the long-tailed property for a specific parameter range, which can lead to exceedingly long runtimes. We also verify this observation with the Glucose 4.1 solver together with Chanseok Oh’s deletion strategy and with MiniSAT [12], a minimalist CDCL solver. This leads to a better understanding of the usefulness of clause deletion techniques in CDCL solvers.

### 1.2 Related work

This section gives a brief overview of related works in both the study of runtime distributions and the research on CDCL solvers.

#### Studying runtime distributions of algorithms

We review previous works, where the runtime distributions of algorithms were studied. In [13], the authors presented empirical evidence for the fact that the distribution of the effort (more precisely, the number of consistency checks) required for backtracking algorithms to solve constraint satisfaction problems randomly generated at the 50% satisfiable point can be approximated by the Weibull distribution (in the satisfiable case) and the lognormal distribution (in the unsatisfiable case). Later, these results were extended to a wider region around the 50% satisfiable point [14]. In [15], the cost profiles of combinatorial search procedures were studied. The authors showed that Pareto-Lévy type heavy-tails often characterize the distributions and empirically demonstrated how rapid randomized restarts can effectively eliminate heavy-tail behavior.

In the paper [11], the hardness distributions of several SLS SAT solvers on logically equivalent modifications of a base instance were studied. The authors included different instance generation models to rule out any influence of the model. Introducing the procedure Alfa that we adapt to CDCL solvers in our work, the paper found that lognormal distributions characterize this hardness distribution perfectly. The approach of [11] lends itself to the analysis of existing SLS-CDCL hybrid solvers, like GapSAT [16]. The advantage of the approach studied in [11] is that the conducted work is not lost in the case of a restart: only the logically equivalent instance could be changed while keeping the current assignment. The paper [17] studied the solvers Sparrow and CCASAT and found that for randomly generated instances, the lognormal distribution is a good fit for the runtime distributions. This study was performed on the domains of randomly generated and crafted instances.

Barrero et al. [18] observed empirical evidence suggesting lognormally distributed runtimes in several types of population-based algorithms like evolutionary and genetic algorithms.

#### Experimental studies on CDCL solvers

The reason behind the fact that CDCL algorithms also incorporate a mechanism to delete (subsets of the) learned clauses from time to time was explained by Mitchell in [19]: Even when sufficient memory is available, the time required to perform unit propagation becomes impractical for extensive clause sets, thus reducing the solver’s performance. Audemard and Simon [10] observed that despite this phenomenon, deleting too many learned clauses can break the learning benefit. Thus, many CDCL solvers let the maximum number of learned clauses grow exponentially. The paper [10] lead to the development of the Glucose solver using “aggressive clause deletion” together with the “Literals Block Distance (LBD)” measure. Some solvers also incorporate a dynamic clause management policy, allowing the solver to *freeze* some learned clauses for later use instead of deleting them [20]. In [21], CDCL solver heuristics such as restarts and clause database management frameworks were analyzed by studying the resolution proofs produced by the solvers.

#### Theoretical studies on CDCL solvers

While our study is purely empirical, it is interesting nevertheless to mention a selection of papers that study CDCL from a theoretical perspective. The field of proof complexity aims at gaining a theoretical understanding of the reasoning power of different proof systems. It is well-known that an implicit CDCL run can be interpreted as resolution proof. In the opposite direction, there has been a line of research [22–24] investigating the proof-theoretic strength of CDCL. This research culminated in papers proving that CDCL (with non-deterministic variable decisions) can efficiently reproduce resolution proofs [25] and CDCL (with random variable decisions) can efficiently find bounded-width resolution proofs [26]. The complexity-theoretic power of restart in SAT solvers was studied in [27].

### 1.3 Organization of this paper

The rest of this paper is organized as follows. First, in Section 2, we introduce the notations of the field of SAT solving that we are using, give a short overview of the technique of conflict-driven clause learning, and provide some statistical background, especially of survival analysis. We proceed to describe the experimental setup in Section 3. Finally, Section 4 investigates whether clause learning is useful on average. Section 5 demonstrates that the runtime distributions of CDCL solver configurations we investigated exhibit a multimodal behavior. This investigation is continued in Section 6, where it is shown that the Weibull mixture distribution is a suitable fit for the runtime distributions of these CDCL solvers.

## 2 Preliminaries

### 2.1 Notation

A *literal* *ℓ* over a Boolean variable *x* is either *x* itself or its negation $\bar{x}≔\neg x$. A *clause* *C* = (ℓ_1_∨…∨ℓ_*k*_) is a (possibly empty) disjunction of literals ℓ_*i*_. If a clause contains only one literal, it is called *unit*. A *CNF formula* $F=C_{1}\wedge \ldots \wedge C_{m}$ is a conjunction of clauses. We also write clauses as a set of literals and CNF formulas as a set of clauses. An *assignment* *α* for a CNF formula F is a function that maps some subset of the variables occurring in F to {0, 1}. If the subset is proper, the assignment is called *partial*, otherwise it is called *total*. By naturally extending *α* by the definition $\alpha \left(\bar{x}\right)≔\bar{\alpha (x)}$, we can define the result of applying *α* to a clause *C*, which we denote by *C*|_*α*_: one deletes all occurrences of literals *ℓ* from *C*, where *α*(*ℓ*) = 0; if there is a literal *ℓ* ∈ *C* with *α*(*ℓ*) = 1, then *C*|_*α*_ = 1. The notation $F{|}_{\alpha }$ denotes the formula where all clauses containing a literal *ℓ* with *α*(*ℓ*) = 1 are deleted, and each remaining clause *C* is replaced by *C*|_*α*_. A clause *C* is called a *logical consequence* of a formula F if, for all assignments *α* with F|_*α*_ = 1, it also holds *C*|_*α*_ = 1. A set *L* of clauses is a logical consequence of F if each clause *C* ∈ *L* is a logical consequence of F. We then call the formulas F and F∪L *logically equivalent*.

### 2.2 Conflict-driven clause learning solvers

*Conflict-driven clause learning* SAT algorithms, or *CDCL* for short, are one of the most remarkable success stories in computer science. Introduced in the works [1, 2], CDCL can yield dramatic speedups over the simple recursive depth-first backtracking approach DPLL [28, 29]. The DPLL algorithm essentially selects an unassigned variable *x* of the formula F it is trying to solve, and branches with calls to DPLL(*F*|_[*x* = 0]_) and DPLL(*F*|_[*x* = 1]_). While CDCL has been intensely studied by theoreticians and practitioners, we still do not have a complete understanding of *all* mechanisms involved.

#### 2.2.1 Clause learning

In the following, we give a simple introduction to one of the most fundamental CDCL techniques: *clause learning*. Informally speaking, this can be seen as a modification of DPLL, where the algorithm adds some clauses to F if it reaches a conflict, i. e., when the partial assignment constructed thus far falsifies a clause in F. The idea behind this is to prune the search tree and avoid having to re-explore some literal assignments that will not lead to a solution.

We introduce clause learning mostly by example, following the exposition in [30], and refer the reader to [3, 31] for more details. As an example, consider as solver input the formula given in conjunctive normal form

$$\begin{array}{c} \begin{array}{cc} & \left(\bar{x_{1}}\vee x_{2}\right)\wedge \left(\bar{x_{2}}\vee x_{3}\vee x_{4}\right)\wedge \left(\bar{x_{2}}\vee \bar{x_{5}}\right)\wedge \left(\bar{x_{4}}\vee x_{5}\vee x_{6}\right)\;\wedge \\ & \left(\bar{x_{7}}\vee x_{8}\right)\wedge \left(\bar{x_{8}}\vee \bar{x_{9}}\right)\wedge \left(x_{9}\vee \bar{x_{10}}\right)\wedge \left(x_{3}\vee \bar{x_{8}}\vee x_{10}\right). \end{array} \end{array}$$ (1)

Suppose that the CDCL solver makes its first *decision* to assign *x*_1_ = 1. The solver always looks out for clauses where all literals but one are assigned value 0 by the current assignment, and the remaining literal is unassigned (so-called *unit clauses*) [3], and assigns this remaining literal so that the clause is satisfied. This process is called *unit propagation* and is repeated until there are no more unit clauses. In our example, using unit propagation, the solver sets *x*_2_ = 1 due to the clause $(\bar{x_{1}}\vee x_{2}){|}_{\left[x_{1}=1\right]}$. It then sets *x*_5_ = 0 because of the clause $\left(\bar{x_{2}}\vee \bar{x_{5}}\right){|}_{\left[x_{1}=1,\;x_{2}=1\right]}$. No more assignments can be made by unit propagation. To move things further along, the solver has to make another decision. In our example, the solver now decides to set *x*_3_ = 0. By unit propagation, *x*_4_ = 1 and *x*_6_ = 1 are assigned. Suppose, in its third decision, the solver sets *x*_7_ = 1. Using unit propagation, the assignments *x*_8_ = 1, *x*_9_ = 0, *x*_10_ = 1, *and* *x*_10_ = 0 are made. This is a *conflict* since the variable *x*_10_ cannot be set to both 0 and 1.

**Fig 1:**
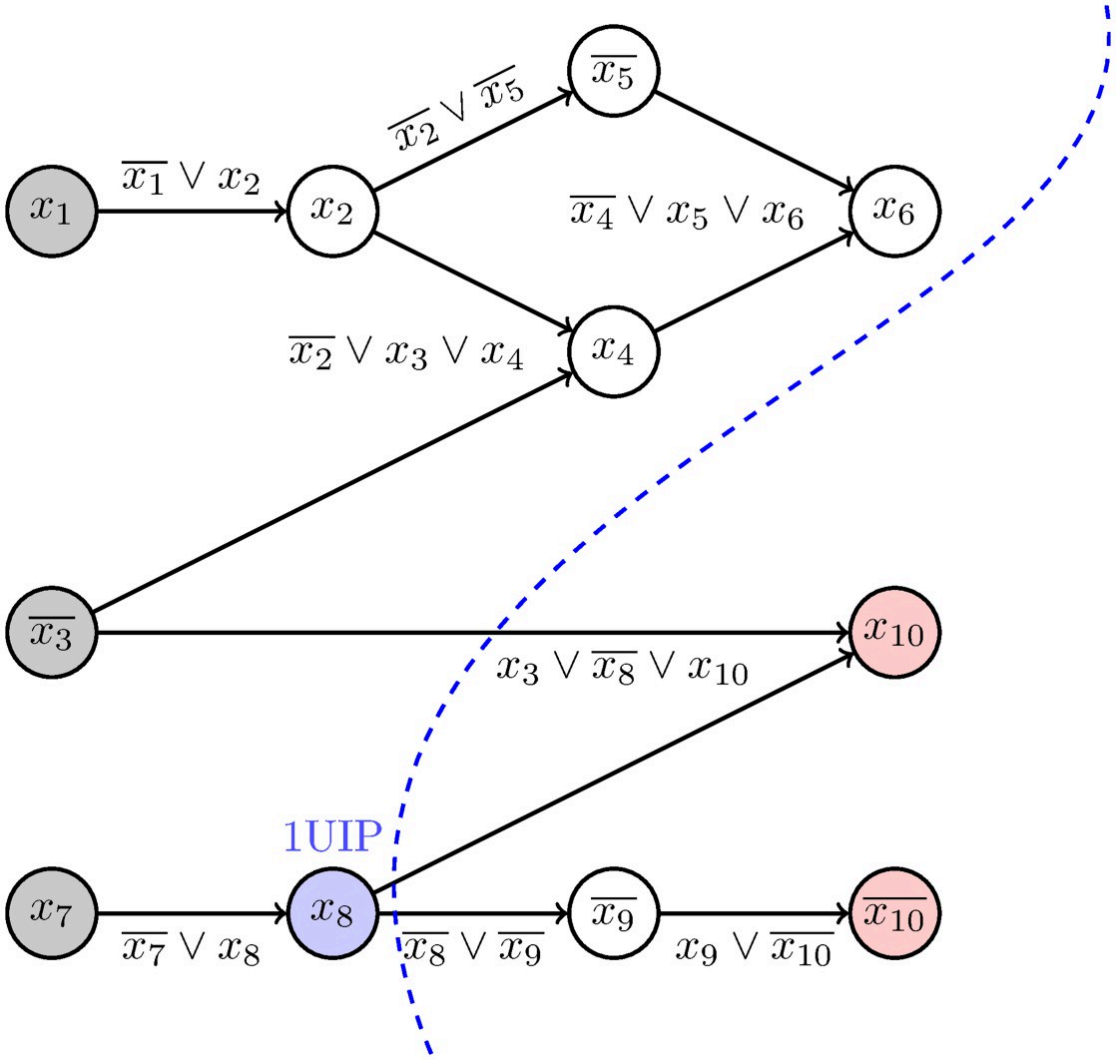
Conflict graph. The figure shows the conflict graph generated in our example run of CDCL when solving formula (1). The decision literals are marked in gray. The conflict literals are marked in red. The reason(s) for a propagation is given as label(s) of the edge(s). The first unique implication point (1UIP) is shown in blue. The 1UIP cut is the dashed blue line leading to the learned clause $\left(x_{3}\vee \bar{x_{8}}\right)$. The graphic is adapted from [30].

During *conflict analysis*, the solver learns a new clause. For this process, the solver uses the *implication graph* that was built in stages during the execution of the algorithm (see Fig 1). In this graph, *decision literals* (in our example, *x*_1_, $\bar{x_{3}}$, and *x*_7_) are the source vertices. The *conflict literals* in our example are *x*_10_ and $\bar{x_{10}}$. Furthermore, the graph includes vertices for every literal that has been assigned the value 1. A directed edge from node *u* to node *v* is included if the value of *v* was set by unit propagation and $\bar{u}$ occurs in the clause that was the *reason* for variable *v* being set.

The *level* of a decision variable *x* is defined as the number of decision variables that have been assigned before *x* plus 1. Literals that are implied by unit propagation by a decision literal *x* have the same level as *x*. In our example, $x_{1},x_{2},\bar{x_{5}}$ have level 1; $\bar{x_{3}},x_{4},x_{6}$ are level 2; and $x_{7},x_{8},\bar{x_{9}},x_{10},\bar{x_{10}}$ are at the *conflict level* 3.

A vertex *v* is called *UIP* (*unique implication point*), if all paths from the conflict level decision literal *x*_7_ to the conflict literals $x_{10},\bar{x_{10}}$ run through *v*. Here, *x*_7_ and *x*_8_ are UIPs. The UIP closest to the conflict literals is called *first UIP* (*1UIP*) [32]. In our example, *x*_8_ is the 1UIP. While there are many ways to learn clauses from conflicts, the most popular method, invoked by a majority of modern CDCL solvers, is based on the 1UIP learning scheme. This learning scheme is considered to work best (see [33] for a comparative study of different learning schemes). In this scheme, the implication graph is uniquely cut such that

1. the 1UIP and all literals assigned before the conflict level are on one side,
2. while all literals assigned after the 1UIP are on the other side (cf. [19]).

This cut, shown in Fig 1, yields the literals $\bar{x_{3}}$ and *x*_8_ as starting points of the separated edges. The clause $\neg \left(\bar{x_{3}}\wedge x_{8}\right)=\left(x_{3}\vee \bar{x_{8}}\right)≕C$ can be shown to be a logical consequence of the original formula. This clause is called the *learned clause*. The solver adds this clause to the clause set.

Using so-called *non-chronological backtracking*, the solver now jumps back to the level of the last variable in *C* being assigned before the variable *x*_8_ of the conflict level, i. e., it jumps back to level 2, where $\bar{x_{3}}$ was assigned. Then, using unit propagation on the clause *C*, the variable *x*_8_ would be assigned 0.

#### 2.2.2 Clause deletion and literal block distance

Many CDCL algorithms also incorporate a mechanism to delete (subsets of the) learned clauses from time to time. As Mitchell explains in [19], this is due to the fact that even when sufficient memory is available, the time required to perform unit propagation becomes impractical for very large clause sets, thus reducing the performance of the solver. However, deleting too many learned clauses breaks down the learning benefit [34]. To identify good learned clauses during the search, [10] introduced the notion of literal blocks. Intuitively, this measure tries to capture semantic relations between literals.

**Definition 2.1** ([10]). Let *C* be a learned clause. Suppose *C* is being partitioned into *k* literal sets with respect to their level. The *literal block distance* (*LBD*) of the clause *C* is defined to be *k*.

Clauses with higher LBD are considered to be less useful. Other measures considered when deleting clauses are the size, age, and activity of a clause [35].

### 2.3 Statistical background

This section briefly introduces the statistical tools used in this paper.

**Definition 2.2** ([36]). Let *X* be a real-valued random variable.

- Its *cumulative distribution function* (*cdf*) is the function F:R→[0,1] with
  $$\begin{array}{c} F_{X}(t)≔R[X\leq t]. \end{array}$$
- Its *quantile function* $Q_{X}:\left(0,1\right)\to R$ is given by
  $$\begin{array}{c} Q_{X}\left(p\right)≔\inf\{t\in R|F_{X}\left(t\right)\geq p\}. \end{array}$$
- If there is a non-negative, integrable function *f*_*X*_ such that
  $$\begin{array}{c} F_{X}\left(t\right)=\int_{-\infty }^{t}f_{X}\left(u\right)\;du, \end{array}$$
  then we call *f*_*X*_ the *probability density function* (*pdf*) of *X*.
- The *survival function* of *X* is given by
  $$\begin{array}{c} S_{X}\left(t\right)≔P_{}[X>t]=1-F_{X}\left(t\right). \end{array}$$

We need the fact that the quantile function is the inverse of the cdf in the next section.

#### 2.3.1 Visual data analysis

To compare two probability distributions, we use the explorative graphical tool of *Q –Q plots*. These plots compare two distributions by plotting their quantiles against each other. If the result is a line, one can assume that the underlying distributions are the same.

**Definition 2.3**. Let *F* and *G* be two cdfs. Then the graph (*F*^−1^(*p*), *G*^−1^(*p*)) for 0 < *p* < 1 is called *Q–Q plot* of *F* and *G*.

*Remark 1* ([37]). If *F* and *G* are identical, the Q–Q plot is the main diagonal. If $F\left(x\right)=G(\frac{x-\mu }{\sigma })$, then *F*^−1^(*p*) = *μ*+ *σG*^−1^(*p*). Thus, the Q–Q plot of *F* and *G* shows a linear relationship of slope *σ* and intersection *μ*.

In a goodness-of-fit problem, one theoretical cdf is given, and we have empirical observations drawn from the other distribution.

**Definition 2.4.** Given a sample *y*_(1)_ ≤ *y*_(2)_ ≤ … ≤ *y*_(*k*)_, we let $p_{i}≔{\hat{F}}_{n}\left(y_{i}\right)$ and *x*_*i*_ ≔ *Q*(*p*_*i*_), where ${\hat{F}}_{n}$ is the empirical cumulative distribution function and *Q* is the theoretical quantile function of a theoretical distribution function *F*. In the *Q–Q plot*, we plot the points (*x*_*i*_, *y*_*i*_) for *i* = 1, …, *k*.

#### 2.3.2 Survival analysis and censored data

We use survival analysis (see [38] for an introduction to the subject) to analyze data in which the time until an event is of interest. The time until this event happens is called *event time*. If all events are observed, we can estimate the cdf with the help of the observations, for which we use the empirical cdf.

**Definition 2.5.** Let *X*_1_, …, *X*_*n*_ be independent, identically distributed real-valued random variables with realizations *x*_*i*_ of *X*_*i*_. Then, the *empirical cumulative distribution function* (*ecdf*) of the sample (*x*_1_, …, *x*_*n*_) is defined as

$$\begin{array}{c} {\hat{F}}_{n}\left(t\right)\frac{1}{n}\sum_{i=1}^{n}1_{\left\{x_{i}\leq t\right\}},\;t\in R, \end{array}$$

where $1_{A}$ is the indicator function of event *A*.

Since in some of our experiments, it turned out to be computationally infeasible to wait until all formula instances are solved, we use a tool from non-parametric statistics to estimate the survival function of the corresponding runtime random variable. That is, we are working with incomplete observations. To nevertheless estimate the survival function from a sample of censored survival data, we use the Kaplan–Meier product-limit estimator [39, 40].

Let *T* be a non-negative random variable (which indicates the time until an event of interest takes place, e. g., finding the solution of a formula). Let *t*_1_, …, *t*_*k*_ be the points in time when events 1, …, *k* would have happened (think of a solution for formula $F_{j}$ being found if the solver was not stopped) whose common distribution is that of *T*. *Right-Censoring* is present when we have some information about event time (e. g., the solver was still running at a certain point in time), but for some events, we do not know the exact event time (because we stopped the solver early). More precisely, to avoid excessively long runtime, we later choose for every *j* ∈ {1, …, *k*} a fixed integer *c*_*j*_ as the *censoring time* for event *j* (meaning that after this time, the solving of $F_{j}$ is aborted). Then, the data available for estimating the survival function *S*_*T*_ of the random variable *T* is the sequence of observations

$$\begin{array}{c} {\left(({\tilde{t}}_{j},c_{j})\right)}_{j=1,\ldots ,k}\;\;\text{with}\;\;{\tilde{t}}_{j}≔\min\left\{t_{j},c_{j}\right\}, \end{array}$$

as well as *censoring indicators* cen_*j*_ ∈ {0, 1} of the form

$$\begin{array}{c} {\text{cen}}_{j}=0:\;\Leftrightarrow t_{j}<c_{j}. \end{array}$$ (2)

That is, we either know that the formula $F_{j}$ was solved in time (and we know the time *t*_*j*_ needed for this), or we know that the solver was still running at the censoring time *c*_*j*_.

**Definition 2.6** ([39, 40]). The *Kaplan–Meier estimator* is given by

$$\begin{array}{c} {\hat{S}}_{T}\left(t\right)≔\prod_{i:\;t_{i}\leq t}(1-\frac{d_{i}}{n_{i}}), \end{array}$$

where (in our case)

- *t*_*i*_ is a point in time when (at least one) formula was solved,
- *d*_*i*_ is the number of experiments, where the solver finished at time *t*_*i*_, and
- *n*_*i*_ is the number of experiments that have not yet had an event or have not been censored up to time *t*_*i*_.

If there are no censored observations, the Kaplan–Meier estimator reduces to one minus the empirical cumulative distribution function (see e. g. [38]), also known as the empirical survival function.

## 3 Experimental setup

There have been significant advances in the theoretical field of proof complexity developing a theoretical understanding of CDCL solvers, as we have surveyed in Section 1.2. Unfortunately, no model completely captures clause deletion in CDCL solvers. For example, in [22, 25, 26], the analyses of theoretical solvers rely crucially on the assumption that the learned clauses are never deleted. For these reasons, an experimental approach seems the most reasonable to investigate the effect of pre-learned clauses and clause deletion.

Let us briefly summarize our experimental approach before discussing its details. First, we recorded all learned clauses L a CDCL solver will find on “its way towards” a solution of a formula F; then, we extended the original instance with subsets L⊆L of these pre-learned clauses; and finally, we analyzed the runtime of such extended instances compared to the original instance F (see Section 3.1 for additional details on and a discussion of this modification process).

We now describe our CDCL solver configurations and choice of benchmarks.

### Investigated solvers and deletion strategies

Our SAT solver of choice for the majority of our experiments was Glucose 4.1 (see [9, 10]) in the non-parallelized version. First introduced in 2009, the Glucose project, which is based on the famous MiniSAT solver [12], was quite successful in the past SAT Competitions. To increase confidence in the results, we also performed experiments using the deletion strategy of Chanseok Oh in combination with the Glucose 4.1 solver. We furthermore extended the examination to the MiniSAT solver.

### Instances

We obtained a relevant, diverse, and well-documented pool of satisfiable and unsatisfiable instances by choosing all instances from the SAT Competition 2020, which were solved by Glucose 3.0 between 30 min and 5000 s (≈ 83 min). We want to remark that Glucose 3.0 was used for the filtering of the instances, however, we later used Glucose 4.1 for our experiments as this was the newest version of the solver that was available at the start of our experiments (this solver did not yet participate in the SAT Competition 2020). The upper bound in the above mentioned time limit comes from the time limit imposed in the SAT Competition, where all solvers are cut off after 5000 s. A more detailed description of all selected instances can be found in S1 Table. We also refer to the proceedings of the SAT Competition 2020 [41, 42]. A vigilant reader may notice that we have 53 instances in our pool, whereas our selection criterion applies to 61 instances of the SAT Competition 2020. We eliminated the remaining eight instances from the pool because they caused technical complications in at least one stage of our experimental setup. For example, three cases failed during clause recording as the number of learned clauses was too high and the required disk space to save all of them exceeded all reasonable limits. On the remaining five instances, Glucose 4.1 ran out of RAM for some extensions. These cases could skew the runtime analysis since we do not know how Glucose 4.1 would have performed with enough memory. Therefore, we excluded them from the analysis.

#### 3.1 Generating extensions from learned clauses

During execution, modern CDCL solvers learn plenty of clauses. All these learned clauses are directly implied by the clauses of the initial formula F, which means that F∪L is logically equivalent to F for all L⊆L, with L being the set of all learned clauses. We call *L* an *extension* of the *base instance* F and F∪L an *extended instance*.

**Algorithm 1. Modified version of a CDCL Solver.** We used this modified version of CDCLSolver ∈ { Glucose 4.1, Glucose 4.1 + ChanseokOh, MiniSAT} in our experiments to model the clause learning and clause deletion process as a random process. Each call of this modified algorithm uses CDCLSolver to solve an extended instance F∪L. This allows us to study the runtime distribution of CDCLSolver.

**Input:** Boolean formula F (the base instance)

Let L be the set of all learned clauses during the execution of CDCLSolver (F)

*L* ≔ ∅

**foreach** C∈L **do**

 with probability *p* **do** *L* ≔ *L* ∪ {*C*}

Call CDCLSolver (F∪L) and record several performance measures.

#### Our model

We adapt the approach presented in [11] to CDCL solvers. We refer to Algorithm 3.1, which requires a pool of pre-learned clauses L. These clauses were gathered by running CDCLSolver ∈ {Glucose 4.1, Glucose 4.1 + ChanseokOh, MiniSAT} on a base instance F and logging all learned clauses to a file. Thus, the set L contains all clauses CDCLSolver learned “on its way” to a satisfying assignment or to an unsatisfiability proof. The random sampling of a subset L⊆L was implemented by independently selecting each clause in the pool L with probability *p*. This subset *L* is used to study the runtime of CDCLSolver on the extended instance F∪L. For our experiments, we chose *p* = 0.01 and generated *N* different extensions *L*^(1)^, …, *L*^(*N*)^ for each of the 53 base instances in our instance pool. That is, for each base instance F, we recorded the performance measures (runtime, number of conflicts, number of propagations, etc.) of CDCLSolver on the extended instances $F\cup L_{\left(1\right)},\ldots ,F\cup LL_{\left(N\right)}$. We chose *N* = 5000 for Glucose 4.1 and *N* = 1000 for the additional experiments with Glucose 4.1 + ChanseokOh and MiniSAT. In this way, we can study the performance of CDCLSolver on instances that *already* contain some of the learned clauses.

The scripts for generating the sets L corresponding to the base instances, as well as the scripts for reconstructing our sampled sets *L* can be found in [43]. Regarding only the experiments where CDCLSolver = Glucose 4.1, we produced 1.5 TB of instance data in the DIMACS format. Recording the performance, we used 265000 calls of Algorithm 3.1 with sometimes surprisingly long runtimes.

#### Interpretation of the model

Our model can be interpreted in the following two ways:

1. We force the investigated solver CDCLSolver to always keep the clauses in *L* in memory, while it is solving F∪L. This is interesting because the clauses in *L* were learned by the same solver on “its way to” a solution. This can be thought of as providing the solver these clauses for free by a *useful* clause oracle.
2. The investigated solver CDCLSolver starts by trying to solve the base instance F. During its execution, the solver learns the clauses of the core pool L. Let a **reset** of the solver be defined as an aggressive clause deletion of part of the learned clauses in the database with a simultaneous deletion of the current assignment. We model this reset as a probabilistic process that takes place just before a solution is found, i. e., the solver deletes each of the learned clauses with probability 1 − *p*. The set L could be thought of as being kept as a *frozen set*. Our model studies the runtime distribution after this reset. See, however, the limitations to this interpretation, as mentioned below.

One should observe that our sampling process was designed so that all extensions have comparable LBD properties. For example, the median LBD of an extension is approximately the median LBD of the pool L. The same holds for various LBD quantiles. Thus, long runtimes on extended instances cannot be explained by the fact that the set *L* of the extended instance has “poor” LBD properties. The same observation holds for the average size of the learned clauses in the extensions. Long runtimes on extended instances are thus not due to the fact that these extensions have a higher number of clauses with more literals and are thus less likely to become unit and actually guide the solver. Additionally, the sizes |F∪L| follow a Gaussian normal distribution for fixed F. Even if a solver scales the size of the learned clause database with the size of the original problem and thus accumulates even more learned clauses, this observation is not enough to explain the multimodality and long-tailed phenomenons observed.

We furthermore would like to mention that we did not increase the base instance F by too many clauses: the median increase of |F∪L| when compared to |F| is just 3%. This increase cannot explain the enormous deteriorations that we have observed.

#### Limitations of our model

One obvious criticism of the approach described above is that our model is not a 100% precise modeling of CDCL solvers as they are implemented in practice. For example, our experimental setup cannot preserve variable activities and phases between the solving of F and F∪L. Our notion of a reset also assumes that the reset takes place just before a solution is found. In practice, a deletion of learned clauses is usually triggered much earlier. However, to conduct a thorough statistical analysis of runtime distributions, we had to make sure that the set L is large enough to sample at least 1000 quite different extensions. Our approach also has the benefit of studying the combined influence of learned clauses from far apart *batches* (a batch is the set of clauses learned between restarts of the solver). Let us mention that a restart of a CDCL solver consists of deleting the current partial assignment while keeping the set of learned clauses. This concept should not be confused with our notion of a reset. We believe that our approach lends itself to getting a more *global* understanding of the role of learned clauses.

While these are valid points, we nevertheless hope that our runtime distribution study provides new insights into the role of clause deletion. The aim of our model is not to completely understand all aspects of CDCL solvers but to get a better understanding of one of them. At the current state of research on CDCL solvers, clearly, all such models will have their drawbacks and will not be able to represent all heuristics correctly. Further points that could be investigated or improved upon in future research are noted in Section 7.

### 3.2 The challenge of solving a myriad of hard formulas

Solving over 265000 hard Boolean formulas in a reasonable time required parallelization, for which we used Sputnik [44], and a somewhat more complex experimental setup. We additionally distributed the formulas over two regular servers (Luna, Erpel) and an HPC cluster (BwUniCluster 2.0). See Table 1 for more details. Note that due to such a diverse hardware setup, runtime comparisons have to be done with caution. More details on this and other metrics can be found in Section 4. During our experiments, we always additionally considered time-independent theoretical measures.

**Table 1:** Summary of the hardware used in our experiments.

Name	Node	CPU	Cores	Frequency	RAM
Erpel		Intel Xeon E5–2698 v3	32	2.30 GHz	256 GB
Luna		AMD EPYC 7742	64	2.25 GHz	256 GB
BwUniCluster 2.0	HPC	Intel Xeon Gold 6230	40	2.10 GHz	96 GB
	HPC Broadwell	Intel Xeon E5–2660 v4	28	2.00 GHz	128 GB

Erpel and Luna are standard server architectures, whereas the BwUniCluster 2.0 is a high-performance computing cluster.

After we initially started the experiments on just the Luna Server, we were confronted with surprisingly long runtimes on certain extended formulas. The corresponding original instances were solved after at most 95 minutes, but for some extended formulas, it took Glucose 4.1 more than ten days to solve them. This led us to

1. distribute the calls of Algorithm 1 over multiple hardware nodes, and
2. introduce a timeout strategy (censoring) as introduced in Section 2.3.2.

Only 12 out of all 53 instances encountered censoring in the Glucose 4.1 experiments, meaning that the solver reached the timeout limit for at least one extended instance. In most cases, the solving of only a few extended instances had to be stopped due to the timeout policy. More details on the number of censored extended instances can be found in S1 Table. Said censoring timeouts *c*_*j*_ were assigned to each extended formula $F_{j}$ according to an offset geometric distribution such that

$$\begin{array}{c} c_{j}∼\text{Geo}(\frac{1}{12\text{h}})+5000\text{s}. \end{array}$$

We have chosen the value of 5000 seconds since this is the cut-off in the SAT Competition. The distribution $\text{Geo}(\frac{1}{12\text{h}})$ has an expectation of 12 hours. This approach led to a total CPU time of 16 years and 8 month. All raw data obtained in this way can be found in [43].

Having used censoring in acquiring our data makes the use of survival analysis, as elaborated in Section 2.3.2, necessary.

## 4 Is clause learning useful on average?

Clearly, augmenting a basic DPLL solver with a clause learning mechanism is far from a modern CDCL solver. However, clause learning is arguably the most important technique in CDCL solvers, lending its name to the paradigm. One would therefore expect that clause learning (especially when guided by state-of-the-art heuristics) is generally useful, or at least in the vast majority of cases, i. e., one would expect that providing the solver with learned clauses for free does increase the performance of the solver when compared to the base instance, where the solver has to learn all clauses by itself (we use interpretation (a) of our model in this section). While individual learned clauses are quite cheap to produce, we provide the solver with a large amount of them. Some of them involved *significant* effort of the solver during generation of the core pool L (deep decision level, complex conflict analysis, etc.), but now these are given for free to the solver.

To check this assumption, we performed a parametric test of whether the mean difference between the base instance and the 5000 (possibly censored) runtimes on the extended instances solved with Glucose 4.1 equals 0. For this, we assumed that the paired differences follow a Gaussian normal distribution. To perform this test, we used the “NADA2: Data Analysis for Censored Environmental Data” package [45] in R (we refer to the book [46] for an in-depth treatment of the statistical methods involved). For the threshold value in the statistical null-hypothesis tests, we used the standard value *p* = 0.05.

In this section, we—quite surprisingly—demonstrate that clause learning is oftentimes useful, but there are also *many* instances where a dramatic negative effect can be observed. We speak of a *negative effect* if the mean of the runtimes required to solve the extended instances was statistically significantly greater (*p* = 0.05) than the runtime required to solve the base instance. We refer to Table 2 for an overview of the different effects. Interestingly, almost all instances can be very clearly categorized in the table because the obtained *p*-values are remarkably low (the exceptions are marked in the table).

**Table 2:** The effect of learned clauses (without deletion) on the runtime of Glucose 4.1.

	With censoring	Without censoring
Positive effect	5	26
No significant effect	0	1 [†]
Negative effect	7	14 [‡]

We say that clause learning has a *positive effect* if the mean of the runtimes required to solve the extended instances was statistically significantly smaller (*p* = 0.05) than the runtime required to solve the base instance. If the mean is statistically significantly greater than the runtime for the base instance, we speak of a *negative effect*. Otherwise, it has *no significant effect*. The base instances are grouped in the table based on whether there was at least one extended instance of this base instance where censoring occurred (*with censoring*) or not (*without censoring*).

⊳ [^†^] The observed *p*-value was *p* = 0.53 for the unsatisfiable instance ncc_none_5047_6_3_3_0_0_41_p0.01 (see S1 Table for the complete list of *p*-values). The observed effect was negative.

⊳ [^‡^] Furthermore, the non-censored instances with negative effects include an instance (namely 6g_5color_164_100_01) with *p* = 0.039. This *p*-value is noteworthy since all other *p*-values are smaller than 2.657 ⋅ 10^−4^ (again, see S1 Table).

It is worth mentioning that in [47], a fundamental difference in the way modern CDCL solvers solve satisfiable and unsatisfiable instances was reported. There is still no consensus in the community regarding the role learned clauses play in obtaining a satisfying assignment. However, most agree that the role of learned clauses is more pronounced when the solver is constructing a proof of unsatisfiability. By analyzing the effect of learned clauses with respect to the satisfiability of the formula, we found that in 63% of the cases, the effect was negative for satisfiable instances and in 27% for unsatisfiable instances (see Table 3).

**Table 3:** The effect of learned clauses (without deletion) on the runtime of Glucose 4.1 with respect to the satisfiability of the base instance.

	Satisfiable	Unsatisfiable
Positive effect	7	24
No significant effect	0	1 [†]
Negative effect	12 [‡]	9

See Table 2 for an explanation of the rows. The dagger-symbols have the same meaning as in Table 2.

We want to emphasize that there are also quite a few instances where adding the set *L* yields a deterioration in the number of conflicts occurring during a run of the solver. In more than 11% of the cases, an adverse effect was observed, which cannot be explained by pure chance and is quite surprising. We refer to Table 4 for an analysis of the effect of learned clauses concerning the number of conflicts. We chose to study this additional measure due to the heterogeneous server architecture outlined in Table 1: The runtimes differ very slightly between the server; however, the number of conflicts needed to solve an instance is a robust, hardware-independent measure.

**Table 4:** The effect of learned clauses (without deletion) on the number of conflicts used by Glucose 4.1.

	With censoring	Without censoring
Positive effect	8	37
No significant effect	0	0
Negative effect	2	4

See Table 2 for an explanation of the rows and columns (replace *runtime* by *number of conflicts*). ⊳ Out of the six instances with a negative effect, two were unsatisfiable, and four were satisfiable. ⊳ Two of the 53 instances (both with censoring) had to be excluded from the tests due to numerical complications in the censored data paired *t*-test (NADA2 package).

For a comparison of runtime vs. number of conflicts, we refer to Table 5. We want to point out that in each case where an opposite effect for time and number of conflicts can be observed, the effect was negative for time and positive for the number of conflicts. As mentioned in Section 1.2, this might be explainable by the observation of Mitchell [19]: the required time to perform unit propagation becomes too high for very large clause sets, which reduces the performance of the solver. If an extended instance already contains more clauses than the base instance, the propagation speed when solving the extended instance is lower. Furthermore, many solvers scale the size of the learned clause database with the size of the original problem, leading to the solver to learn even more clauses, which causes the propagation speed to lower again. Another factor in play is that when the restart duration is high, the learned clauses are often larger on average, compared to frequent short restarts. These large learned clauses take more time to produce (they have deeper decision levels, take more propagations to reach, and require a more complex conflict analysis, etc.).

**Table 5:** Comparison between the effect of adding clauses (without deletion) for time and number of conflicts of Glucose 4.1.

	With censoring	Without censoring
Same effect for time and number of conflicts	7	30
Opposite effect	3	10

We excluded one instance where, for at least one measure, our experiments could not determine if there was a positive or negative effect for that measure.

In both cases, i. e., regardless of whether one considers the runtime or the number of conflicts, the observed performance deterioration cannot be explained by pure chance. This is all the more surprising since the set of learned clauses *L* is not just any random set of clauses but clauses that the same solver learned on “its way to” a solution. These clauses should thus contain very useful information for the solver when it is solving the same formula from scratch but with this additional information. Therefore, one would expect that each and any of such a clause would benefit the guidance of CDCL towards a solution.

To explain this deterioration phenomenon, one should therefore consider the influence of clause *deletion*. Our experimental setup can be interpreted as switching off clause deletion for the set of added clauses *L* (while keeping all other heuristics and optimizations of the solver) and learning all those clauses at once. Note that the solver learns some additional clauses during its run and can delete them. The set *L*, however, is fixed during the run. Seemingly, clause deletion at the right points in time is as crucial as clause learning. This statement cannot be fully explained by a blow-up in the size of the clause database, as the unit-propagation-independent measure of the number of conflicts also increased in many cases.

## 5 Multimodal behaviors in CDCL solvers

In Section 4, our focus was to compare the behavior of Glucose 4.1 on the unmodified base instance to the behavior on the modified instances that extended this base instance. From now on, we focus on studying the distributions of the modified instances with respect to different measures (see interpretation (b) of our model).

Our precise aim in this section is to get an understanding of the modality of the ensuing CPU time distributions. Recall that in statistics, a probability distribution with a single peak is called *unimodal*; otherwise, we speak of a *multimodal* distribution (a more formal definition can be found in [48]). An easy way to inspect the modality of a distribution is to inspect the histogram of the distribution visually. This method has the additional advantage that no statistical test has to be used that can distinguish between unimodal and bimodal distributions but rely on the knowledge of the underlying distribution type (i. e., one does not need to know in advance if the distribution can be resolved into, e. g., *normal* distributions [49]).

Since our obtained data points are censored, we cannot immediately plot the histogram. To overcome this obstacle, we have used the Kaplan–Meier estimator (see Definition 2.6) implemented in the Survival package [50] in R to obtain a fit of the underlying survival function. The Kaplan–Meier fit can be computed without prior knowledge of the underlying distribution. Graphically, the Kaplan–Meier survival curve is a step function with a drop each time the solver has finished an instance. The points where a drop can be observed can thus be used as an estimation basis to create the histogram. Note that in the improbable event that two instances take precisely the same time to be solved (while solving times are often very large), the resulting histogram underestimates the number of instances in the corresponding bin. However, this kind of event occurs so seldom that, for all intents and purposes, we can be satisfied with the obtained estimation of the actual histogram.

For a more detailed investigation of multimodal behavior, we have used Hartigans’ dip test statistic [48]. This test determines the amount of multimodality by “the maximum difference, over all sample points, between the empirical distribution function, and the unimodal distribution function that minimizes that maximum difference” [48]. This notion is presented more formally in the following definition.

**Definition 5.1** ([48]). Let

$$\begin{array}{c} \rho \left(F,A\right)≔\underset{G\in A}{\inf}{∥F-G∥}_{\infty } \end{array}$$

for any function space A of bounded functions. The *dip* of a cdf *F* is given by

$$\begin{array}{c} D(F)≔\rho (F,U), \end{array}$$

where U is the class of unimodal distribution functions.

Phrased differently, the dip of a distributions function measures departure from unimodality. We have used a threshold dip value of 0.005 to classify multimodal histograms: All histograms with a dip value over 0.005 can clearly be seen to exhibit multimodal behavior, using only visual inspection. We want to emphasize that this cut-off value is rather strict (see e. g., Fig 4(b) which is clearly multimodal but below the threshold value).

**Fig 2:**
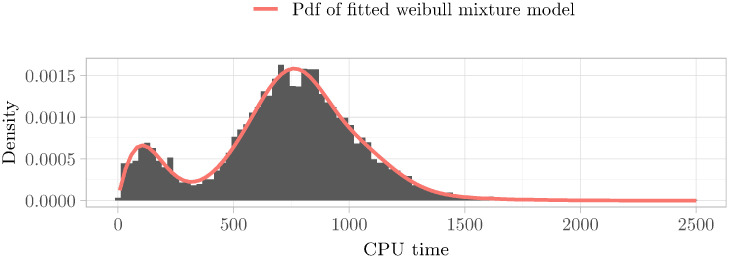
Multimodal histogram of runtime distribution. We used the Kaplan–Meier estimate to obtain the histogram of the runtime distribution of the instance UNSAT_ME_seq-sat_Thoughtful_p11_6_59-typed.pddl_43. We used the *Expectation–maximization* (EM) method to obtain the pdf of the fitted Weibull mixture model (see Definitions 6.1 and 6.2 for an introduction to this kind of distribution). The EM algorithm is an algorithm that allows cluster analysis by starting with a heuristically initialized model and alternating between two steps. First, in the expectation-step (*E-step*), the association of the data points to the different clusters gets changed. Then, in the maximization-step (*M-step*), the model’s parameters get improved by using this new association of the data points. We refer to the classic paper [51] for an introduction to the algorithm. The resulting fitted distribution that is seen in the plot is clearly multimodal. This is supported by a Hartigans’ dip test value of 0.015 > 0.005.

We printed the resulting estimated histogram using Kaplan–Meier of a representative instance in Fig 2. As can be clearly seen, the distribution is multimodal, as witnessed by the two peaks. The Hartigans’ dip test value also confirms this.

**Fig 3:**
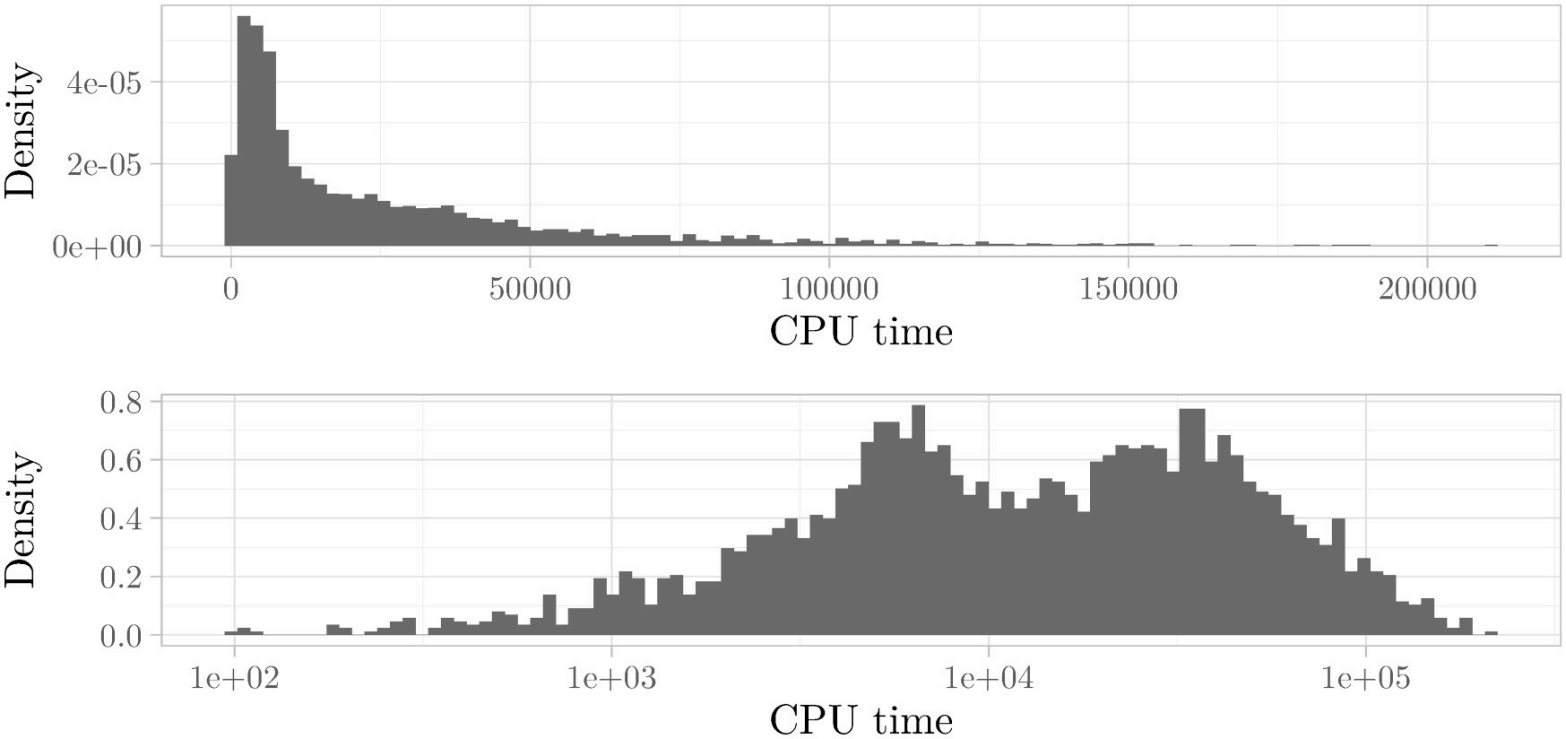
Histogram of runtimes vs. histogram of logarithmically scaled runtimes. Scaling the *x*-axis of a histogram logarithmically can often uncover multimodality that is not clearly visible in the unscaled histogram. The graphic depicts both histograms for the instance size_5_5_5_i019_r12, where this difference is very pronounced. **(above)** Histogram of CPU times with a Hartigans’ dip test value of 0.005. **(below)** Histogram of logarithmically scaled CPU times with a Hartigans’ dip test value of 0.016.

To facilitate our inspection of the histograms, we also investigated the histograms for the logarithmically scaled runtimes. This method has been found to usually give a clear separation into a visible multimodal histogram if the underlying distribution is indeed multimodal (see e. g. [52, 53] for the earliest applications of this technique). In Fig 3 we have shown an instance where the multimodality cannot be seen in the histogram at first glance but can clearly be made out in the histogram with logarithmically scaled runtimes. Using this technique, we found that a significant fraction of 25% of the instances exhibited dominant multimodal behavior.

**Fig 4:**
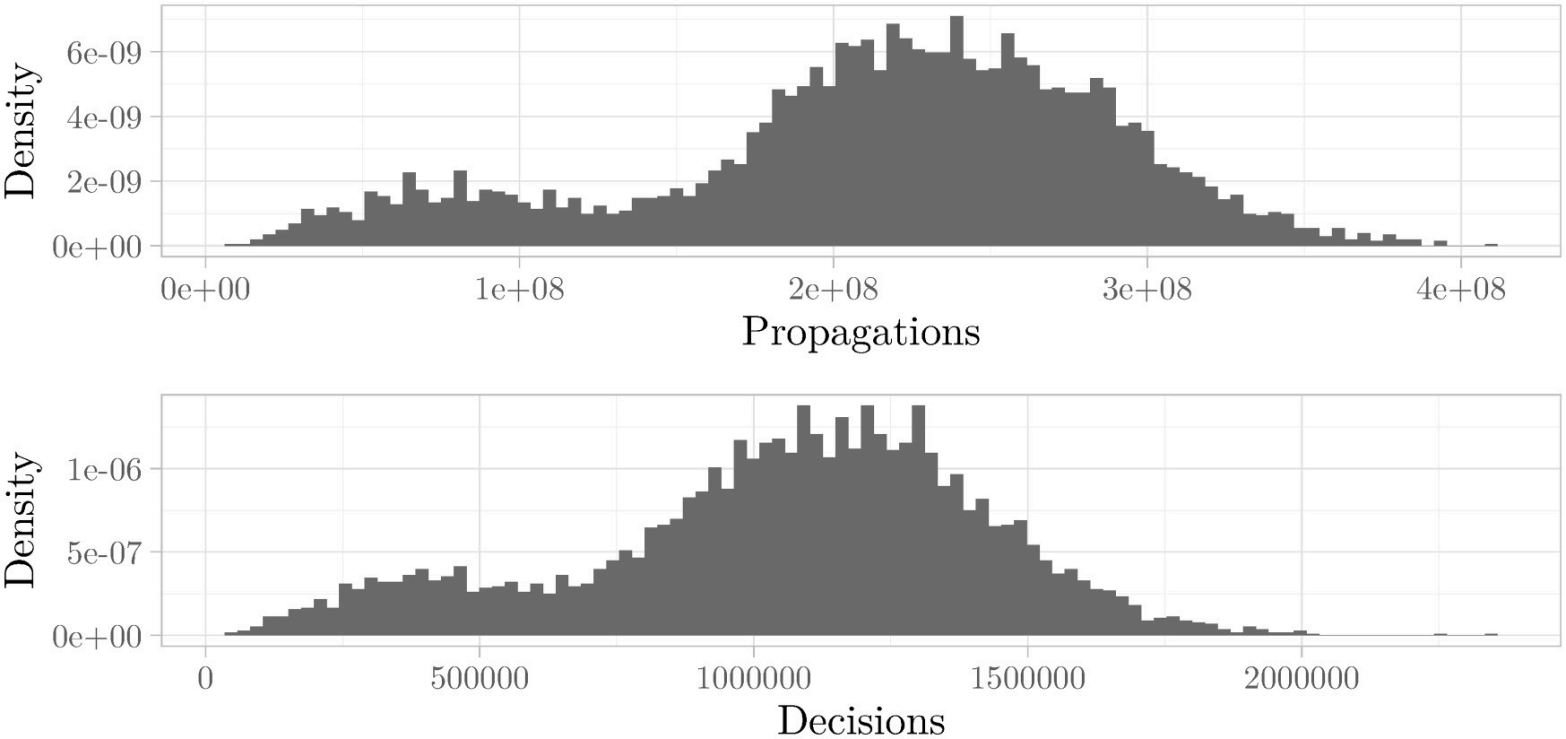
Multimodal histogram of the distribution for the number of propagations and decisions. The histograms show the distribution of the two measures propagations and decisions required to solve the extended instances of UNSAT_ME_seq-sat_Thoughtful_p11_6_59-typed.pddl_43. Both histograms for the hardware-independent measures have the same multimodal form as the histogram for CPU time shown in Fig 2. **(above)** Histogram for number of propagations with a Hartigans’ dip test value of 0.005. **(below)** Histogram for number of decisions with a Hartigans’ dip test value of 0.004.

Due to the heterogeneous server architecture, we also studied the histograms for hardware-independent measures, like the number of propagations and decisions needed to come to a solution. Again, we could observe the multimodality phenomenon for these measures. We refer to Fig 4 as a continuation of the histogram shown in Fig 2.

Using the threshold value of 0.005 for Hartigans’ dip test, 32% of all instances exhibit multimodal behavior for at least one measure (CPU time, logarithmized CPU time, propagations, or decisions).

This multimodal grouping of instances into several categories could be helpful in an investigation of the usefulness of the added clauses. We make this thought more precise in the next section and investigate the distribution type underlying the model.

## 6 Finding the right mixture distribution type

In Section 5, we have already seen that the runtime behavior is multimodal for a substantial part of the instances. This section aims to study which types of distributions are suitable to describe this behavior. Since most well-known distributions, such as the normal distribution, are unimodal or at most bimodal, this suggests that one must resort to another type of distribution.

The presence of the many peaks indicates that the different extended instances (and underlying clauses) can be divided into categories. Each category corresponds to the hardness of the extended instance, where the hardness is again not a fixed value but a random variable. If one confines oneself to a single category of extended instances, one is (potentially) no longer confronted with multimodal behavior but can describe the remaining data utilizing a unimodal distribution.

By (randomly) adding the clauses to *L*, we then end up in this category of extended instances with a certain probability. If such an analysis is conducted for each category, we eventually obtain a description of the complete runtime behavior. Specifically, this means that for each category, the underlying runtime distribution, as well as the probability of ending up in that category, must be identified. The runtime behavior across all extended instances is then characterized by a so-called finite mixture distribution.

**Definition 6.1**. Let *X* be a random variable having the cdf *F*_*X*_. Let *F*_1_, *F*_2_, …, *F*_*N*_ be cdfs and *p*_1_, *p*_2_, …, *p*_*N*_ be weights with *p*_*i*_ > 0 for all *i* ∈ {1, …, *N*} and $\sum_{i=1}^{N}p_{i}=1$. If

$$\begin{array}{c} F_{X}\left(x\right)=\sum_{i=1}^{N}p_{i}\cdot F_{i}\left(x\right) \end{array}$$

holds for all x∈R, then *X* has an *N-component (finite) mixture distribution*.

**Fig 5:**
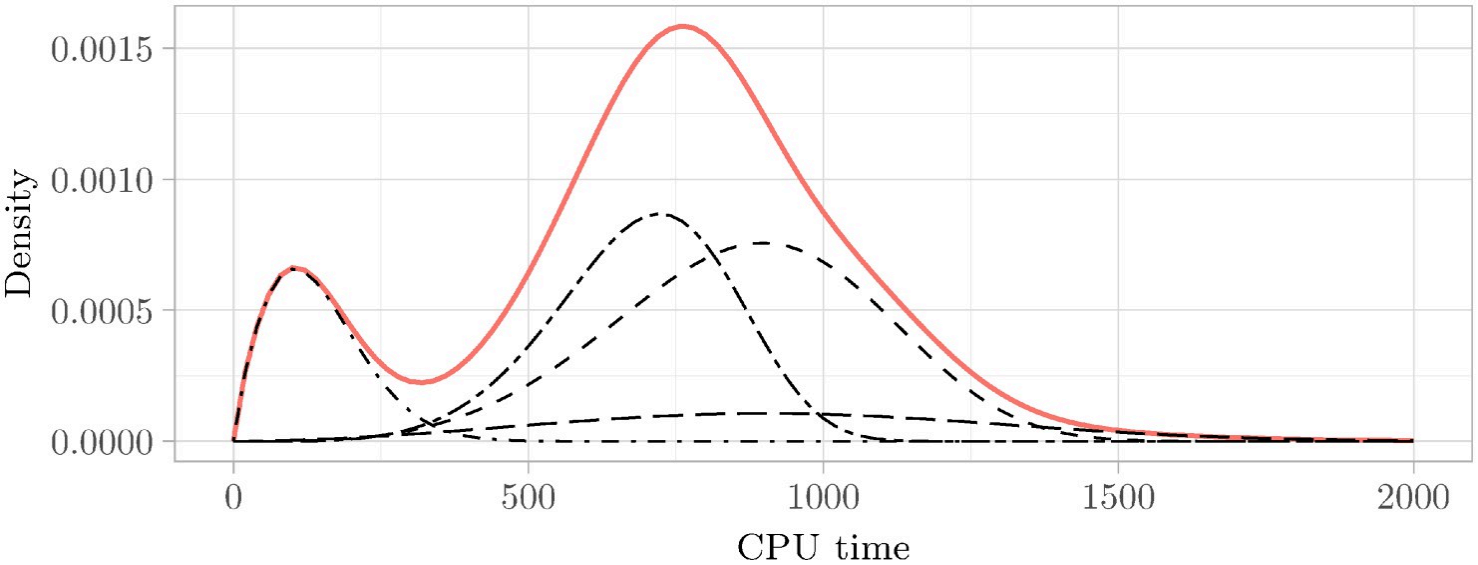
Mixture distribution and components. The figure shows the Weibull components (in black) underlying the mixture distribution (in red) of instance UNSAT_ME_seq-sat_Thoughtful_p11_6_59-typed.pddl_43. The Weibull components were scaled according to their respective *p*_*i*_-values. For the histogram of this instance, refer to Fig 2.

In our case, *N* can be understood as the number of categories. Furthermore, *p*_*i*_ describes the probability of ending up in category *i*, in which case *F*_*i*_ is the runtime distribution for category *i*. We refer to Fig 5 of a depiction of the components underlying a mixture distribution.

In principle, one can choose arbitrary cdfs *F*_1_ to *F*_*N*_. However, it is common practice to choose the cdfs from the same family of (parametric) distributions, where only the parameters of the distribution differ. For example, a popular model are Gaussian mixture distributions in which the cdfs describe normal distributions varying with respect to their expected value and variance. Therefore, the question arises of which parametric distribution type should be chosen for the mixture distribution. We shall argue that a distribution based on the so-called Weibull distribution is an appropriate type of distribution.

**Definition 6.2** ([54]). A random variable *X* with a pdf given by

$$\begin{array}{c} f_{X}\left(x\right)=\{\begin{array}{cc} \frac{k}{a}\cdot {\left(\frac{x-\ell }{a}\right)}^{k-1}\cdot e^{-{\left(\frac{x-\ell }{a}\right)}^{k}}, & x\geq 0\\ 0, & x<0 \end{array} \end{array}$$

is *3-parameter Weibull distributed* with parameters $k\in R^{+}$ (*shape*), $a\in R^{+}$ (*scale*), and ℓ∈R (*location*). The cdf of *X* is given by

$$\begin{array}{c} F_{X}\left(x\right)=\{\begin{array}{cc} 1-e^{-{\left(\frac{x-\ell }{a}\right)}^{k}}, & x\geq 0\\ 0, & x<0. \end{array} \end{array}$$

If the location parameter *ℓ* is zero, we call the distribution *2-parameter Weibull distributed* or just *Weibull distributed*.

We first start by analyzing instances that can be described with only one component (i. e., a 1-component mixture distribution). The idea behind this is that one can derive information about the instances that require more than one component. A suitable graphical tool for this analysis is provided by Q–Q plots, where the observed quantiles are plotted against the theoretical quantiles of a given distribution (recall Section 2.3.1). In the following, we consider the required CPU time until the respective instance is solved, i. e., either a satisfying assignment is constructed, or a proof of unsatisfiability is established.

**Fig 6:**
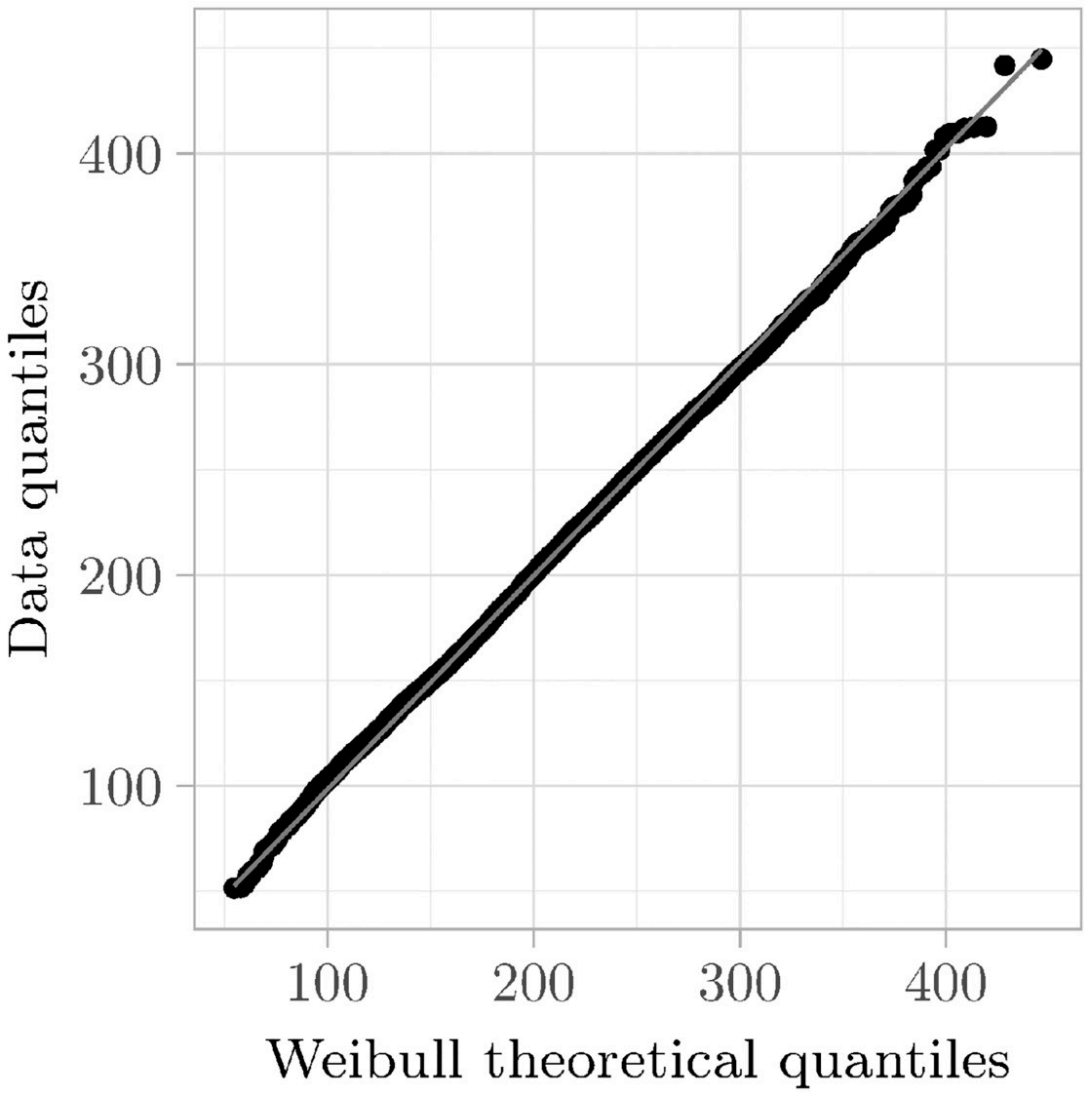
Q–Q plot. The Q–Q plot for instance crafted_n11_d6_c4_num19 was obtained by the quantiles of a fitted 3-parameter Weibull distribution and the data quantiles. The plot appears as a straight line. The correlation coefficient calculates to 0.9997979. For reference, the identity is given in gray.

As an example, we consider the data from one instance in Fig 6. We use a fitted 3-parameter Weibull distribution as the theoretical distribution. As can be seen in the figure, the Q–Q plot yields a straight line, indicating that the theoretical distribution can describe the empirical data very well. So we can conclude that a 3-parameter Weibull distribution is a suitable description for this instance. This is not only the case in this example. For example, one can use the correlation coefficient to measure how linear a certain relationship is. A correlation coefficient of 0.999 describes an extremely strong linear relationship. We used this value and examined all Q–Q plots. In total, 12 instances reach a correlation coefficient of at least 0.999 if a fitted 3-parameter Weibull distribution is used as theoretical distribution. This suggests that a substantial number of instances can be described by a *single* Weibull distribution. It should also be emphasized that other typical distribution types, such as the normal or lognormal distribution, do not provide good fits. While Weibull distributions describe a considerable fraction of the instances, this begs the question of what to do with the remaining instances.

First, it should be emphasized that especially the instances where multimodality is strong cannot be described by a single Weibull distribution. Then, however, it is natural to assume that the individual components of a mixture distribution follow Weibull distributions. We pursue this line of thought in more detail in the following.

Graphical analyses are well suited to argue that Weibull distributions are appropriate. First, we examine how the Weibull distribution behaves at the left tail, i. e., the behavior if *x* approaches 0. It is well known that Weibull distributions have a linear appearance on a log–log plot of the cdf at the left tail. To see this, we investigate the logarithm of the Weibull cdf *F* with location parameter *ℓ* = 0 for *x* ≥ 0:

$$\begin{array}{c} \text{log}F\left(x\right)=\text{log}(1-e^{-{\left(\frac{x}{a}\right)}^{k}}). \end{array}$$

Plugging the Taylor expansion of exp(−*x*) into this equation yields:

$$\begin{array}{cc} \text{log}F(x) & =\text{log}(1-[1-{\left(\frac{x}{a}\right)}^{k}+\frac{{\left(\frac{x}{a}\right)}^{2k}}{2!}-\cdots ])\\ & =\text{log}({\left(\frac{x}{a}\right)}^{k}-\frac{{\left(\frac{x}{a}\right)}^{2k}}{2!}+\cdots ). \end{array}$$

Considering the behavior as *x* approaches 0, we notice that the trailing terms approach 0 much faster than (*x*/*a*)^*k*^ and thus can be neglected. Hence, we obtain:

$$\begin{array}{c} \text{log}F\left(x\right)\approx \text{log}({\left(\frac{x}{a}\right)}^{k})=k\cdot \text{log}x-k\cdot \text{log}a\;\text{for}\;x\to 0. \end{array}$$

By substituting *z* = log*x*, one finds that the cdf *F* indeed appears linearly in the neighborhood of zero on a log–log plot.

For mixture distributions, this method is useful for making statements about the smallest component. Suppose that $F\left(x\right)=\sum_{i}^{N}p_{i}F_{i}\left(x\right)$ is the cdf of a mixture distribution. Here, *F*_1_ is the cdf of a Weibull distribution, and for small *x*, we have *F*_1_(*x*)≫0 and *F*_2_(*x*)≈*F*_3_(*x*)≈…≈*F*_*N*_(*x*)≈0. Thus, *F*(*x*)≈*p*_1_*F*_1_(*x*) is also valid; moreover, due to the reasoning above, the cdf *F* appears linearly on a log–log plot in the neighborhood of zero. Conversely, one can argue that log–log plots of the cdf are suitable for evaluating whether the smallest cdf can be characterized by a Weibull distribution.

Another popular method of analyzing Weibull distributions is to examine the survival function *S*(*x*) = 1 − *F*(*x*). In particular, the survival function transformed as follows is used:

$$\begin{array}{cc} \text{log}(-\text{log}S(x)) & =\text{log}(-\text{log}e^{-{\left(\frac{x}{a}\right)}^{k}})\\ & =\text{log}({\left(\frac{x}{a}\right)}^{k})=k\cdot \text{log}x-k\cdot \text{log}a. \end{array}$$

In other words, a Weibull distribution appears linear if the survival function is double logarithmized in this manner and the *x*-axis is singly logarithmized. We can apply this graphical tool to determine whether the largest component in a mixture distribution can be described by a Weibull distribution.

Again, suppose that $F\left(x\right)=\sum_{i}^{N}p_{i}F_{i}\left(x\right)$ is the cdf of a mixture distribution. Here, *F*_*N*_ is the cdf of a Weibull distribution, and for large *x*, we have *F*_*N*_(*x*)≪1 and *F*_1_(*x*) ≈ *F*_2_(*x*) ≈ … ≈ *F*_*N*−1_(*x*) ≈ 1. Thus, we have

$$\begin{array}{cc} S\left(x\right)=1-F\left(x\right)=1-\sum_{i=1}^{N}p_{i}F_{i}\left(x\right) & \approx 1-\underset{=1-p_{N}}{\underset{︸}{\sum_{i=1}^{N-1}p_{i}}}-p_{N}F_{N}\left(x\right)\\ & =p_{N}-p_{N}F_{N}\left(x\right)=p_{N}(1-F_{N}\left(x\right)). \end{array}$$

By the above argument, the doubly logarithmized survival function *S* and singly logarithmized *x*-axis will appear approximately linear for large *x*. Conversely, such a plot can also be used to deduce whether the largest component can be described by a Weibull distribution.

These two plot types are therefore suitable for finding out whether the extreme values, i. e., particularly short and particularly long runs, are described by Weibull distributions, respectively. Thus, as before, we examine the CPU times and investigate them with the help of these two plot types.

**Fig 7:**
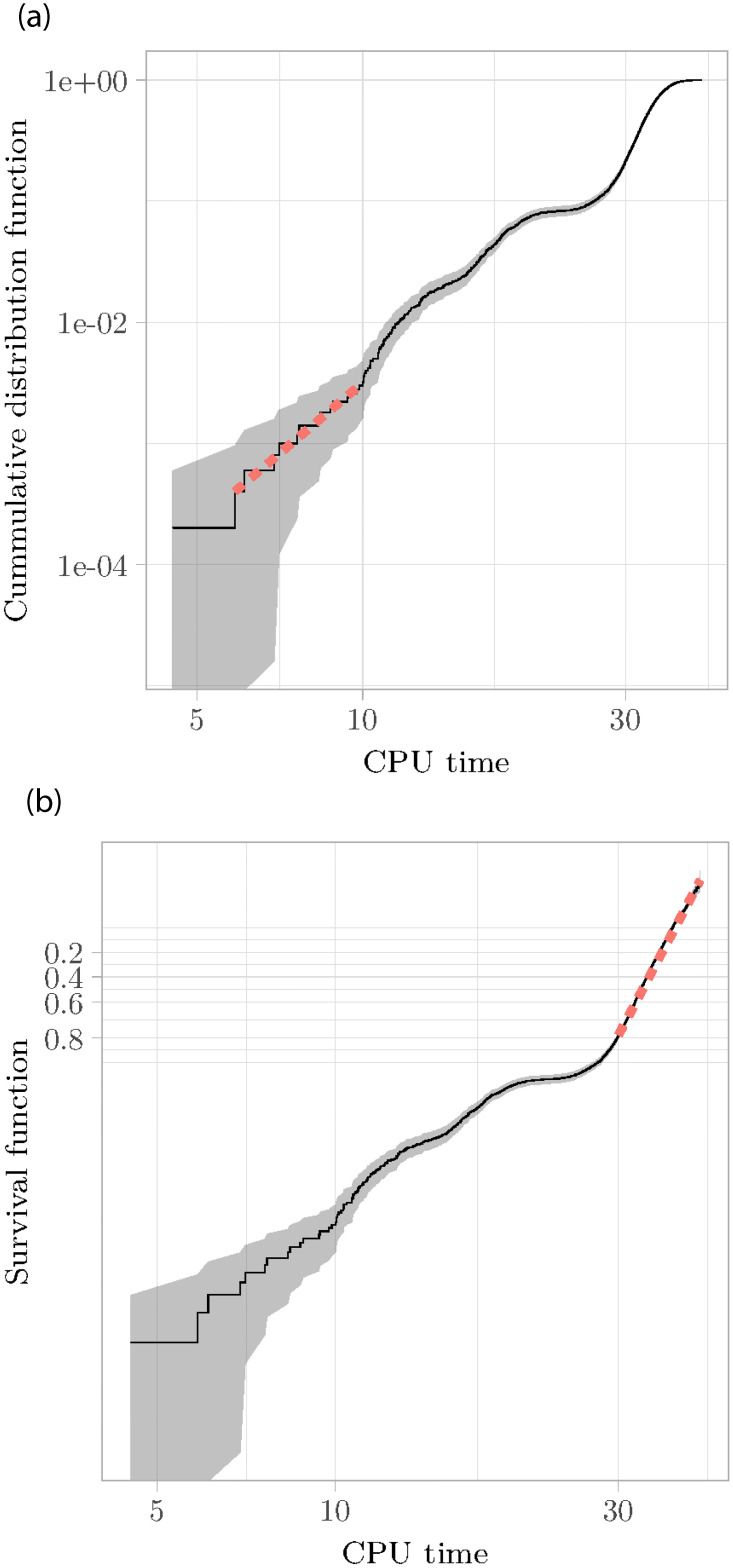
Inspection of the smallest and largest component of the Weibull mixture model. Based on the Kaplan–Meier estimator, the estimations of the cdf and survival function of the multimodal instance bivium-40–200 are shown (Hartigans’ dip test value 0.010). Both the left and the right tails appear as straight lines (depicted in red). The plot of the cdf is a log–log plot, while the plot of the survival function is a loglog–log plot. The gray area marks the confidence interval. This suggests that the smallest and largest component of the underlying mixture model are Weibull distributions. **(left)** Estimation of the cdf. **(right)** Estimation of the survival function.

In Fig 7, we exemplarily consider one instance. Note that both the left and the right tails appear as straight lines. Using the reasoning presented above, we can therefore infer that for both cases, a Weibull distribution is appropriate to characterize the left and the right tail, respectively. On the one hand, Weibull distributions describe both the left and the right tail and, in some cases, the entire support. On the other hand, it is common practice to use only one type of distribution for mixed distributions. Therefore, we argue that the runtime distributions can be described by Weibull distributions.

One can derive some highly intriguing insights into the operation of CDCL solvers from the knowledge that Weibull distributions describe the runtime behavior of such solvers. First, if the shape parameter *k* of the Weibull distribution is less than 1, then the distribution has the so-called long-tail property [55] (for *k* = 1, the Weibull distribution reduces to an exponential distribution which is *light-tailed*, and thus not long-tailed. For *k* > 1, the Weibull distribution is also light-tailed [55]).

**Definition 6.3** ([55, 56]). We say that a positive, real-valued random variable *X* is *long-tailed*, if and only if for all $x\in R^{+}$ it holds $P_{}[X>x]>0$, and for all $y\in R^{+}$ it holds $\lim_{x\to \infty }P_{}[X>x+y]/P_{}[X>x]=1$.

**Fig 8:**
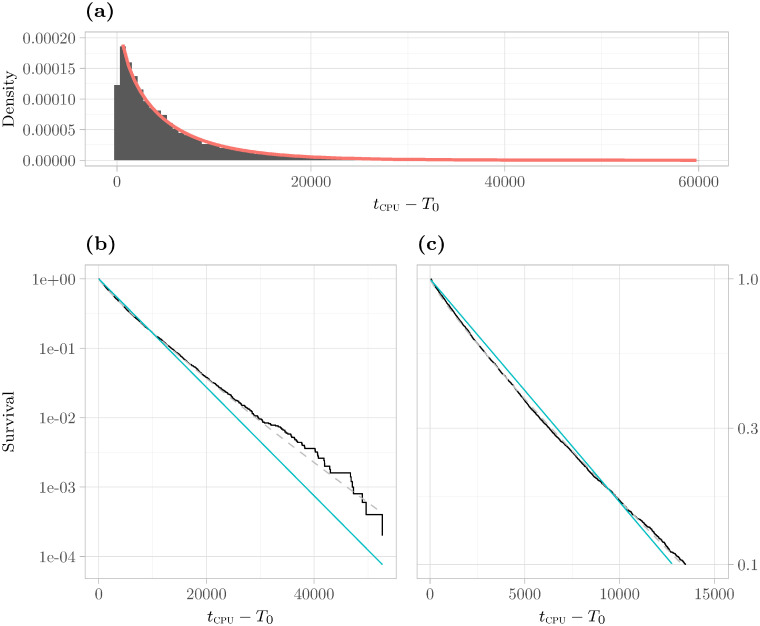
Long-/Heavy-Tails. This figure shows various plots of the unimodal instance 6g_5color_164_100_01 (Hartigans’ dip test value 0.003). This is an example of an instance with a long-tailed runtime distribution. **(a)** The plot shows the histogram of runtimes (in gray) and the fitted pdf (in red). Both are shifted to the left by the minimal time *T*_0_ required to solve any extended instance. The obtained shape parameter of the fit is *k* = 0.884 < 1. Thus, the distribution is long-tailed. **(b)** We have plotted the logarithm of the tail of the distribution, i. e., log*S*(*x*). By visual inspection, one can see that it decays sub-linearly. In this case, ${\text{lim}\;\text{inf}}_{x\to \infty }-\text{log}P_{}[X>x]/x=0$. This property characterizes the class of so-called *heavy-tailed* distributions (a superset of the class of long-tailed distributions) [55]. Intuitively, this means that the algorithm has a non-vanishing probability of requiring very long runtimes. For comparison, we have plotted the logarithmic survival function of an exponentially distributed random variable with the same expectation in blue. The logarithm of the tail of such an exponential distribution decays linearly. **(c)** Zoomed in version of (b). This clearly shows the sublinear decay by focusing on the curvature.

Roughly speaking, this property indicates that the algorithm either finishes (relatively) quickly or takes exceedingly long. We want to remark that long-tails are not equivalent to heavy-tails or powerlaws. For an illustration of a long-tailed runtime distribution and a further elaboration on the subject of the long-tailed property, refer to Fig 8. See also Fig 3(a) for another depiction of a long-tailed distribution.

**Fig 9:**
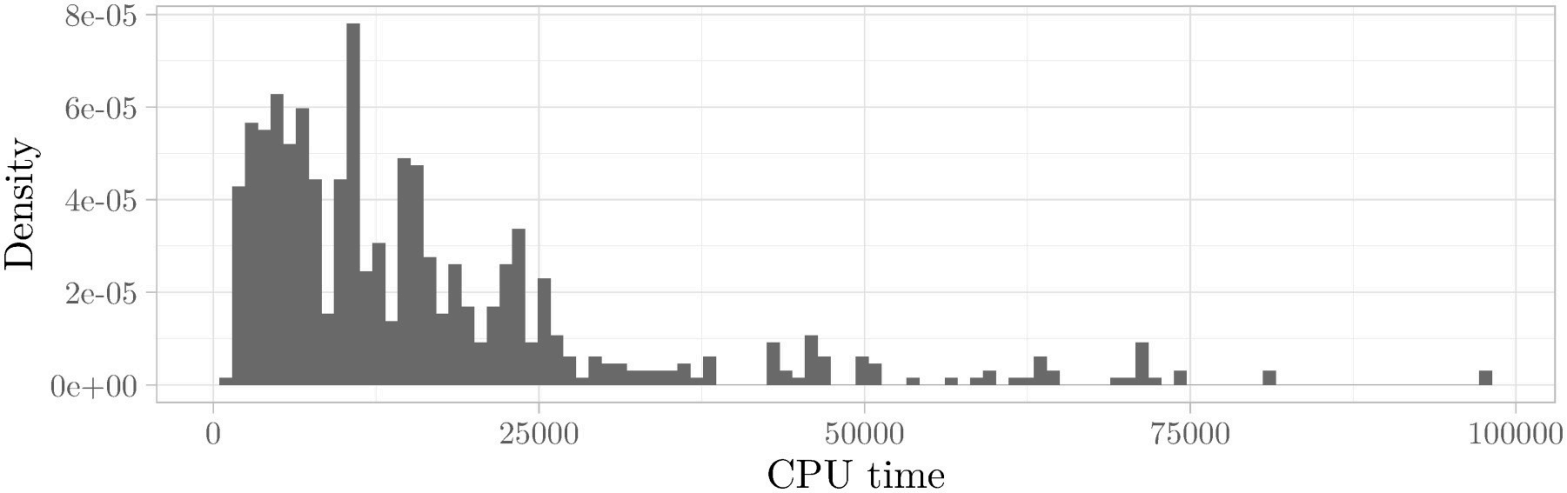
Multimodal and long-tailed effect with MiniSAT. The histogram shows the distributions of CPU times when solving the extended instances of 6g_5color_164_100_01 with MiniSAT. Both a multimodal behavior as well as a long-tailed effect are visible. The multimodality is confirmed by a Hartigans’ dip test value of 0.031.

We have also conducted experiments with Glucose 4.1 using the deletion strategy of Chanseok Oh. Again, there are long-tails present. The same holds for experiments conducted with the MiniSAT solver (where the mean runtimes worsened significantly when compared to Glucose 4.1). We refer to Fig 9.

What is remarkable about this is that algorithmic restarts have been proven to be useful for long-tailed distributions [11, 57]. This means that the algorithm can be accelerated by reinitializing it from scratch. In our context, such a reset of the solver consists of discarding all added clauses *L* that were added to the original base instance F (we, again, would like to point out that such a reset is not the same as a restart in a CDCL solver). Instead, a new set of clauses *L*′ is sampled from the base set of frozen clauses L that is then added to the original instance F. Of course, the search tree is also reset to the top level at the same time.

Therefore, the observation that Weibull distributions describe the runtime behavior implies that aggressive clause deletions (in the form of complete database flushes) together with forgetting the partial assignment are useful in the context of CDCL solvers, i. e., they improve the runtime. However, what is remarkable about this is not the mere observation that these two techniques improve the runtime because this fact has already been shown empirically (see e. g. [2, 10, 58, 59]). It is more interesting that we are reaching a conclusion as to **why** these techniques have a positive effect on the performance. Adding new clauses to the base instance has the inherent effect of making runtimes long-tailed. While the added clauses usually improve the performance, there is a non-negligible chance that the performance deteriorates (sometimes drastically). The easiest way to circumvent this problem is to delete the learned clauses and reset the search tree periodically.

It is also worth repeating the observations on Table 4. Here, the effect of adding clauses is measured by the number of conflicts. This table tells us that, contrary to common belief [19], the degraded performance is not only due to the increase in the size of the base instance F and thus due to a more considerable overhead for each propagation. Instead, it implies that some clauses lead the CDCL algorithm itself astray, i. e., to a path in the search tree that does not yield a solution.

Since clause deletions and forgetting partial assignments are useful because Weibull distributions describe the runtimes, this shifts the question to why adding clauses causes Weibull distributions. A possible starting point is the Fisher–Tippett–Gnedenko theorem [60–63]. Roughly speaking, this theorem states that the minima and maxima of independent and identically distributed random variables converge to one of three distribution types (under the condition that they converge at all). The Weibull distribution is one of these three distribution types. This suggests that the reason for the observed runtimes may be a minimum or maximum process. For example, the runtimes could be significantly influenced by the quality of the “best” or “worst” clause, where by the quality of the clause, we mean the extent to which the clause guides the heuristics of the CDCL algorithm towards a solution. However, this is only a hypothesis that should be further investigated in other future research.

## 7 Conclusion and further research

We have modeled the technique of clause learning in CDCL solvers by solving new logically equivalent formulas of a base instance. This allowed us to analyze the resulting runtime distribution.

We have provided compelling evidence that this distribution is a Weibull mixture model, completing the runtime distribution study [11] for both paradigms of SAT solvers. In addition, the Weibull fit was suitable for both multimodal and unimodal instances. Because the underlying distribution is Weibull, adding new clauses thus has an inherent effect of making runtimes long-tailed in both SLS and CDCL solvers. The long-tailed runtime distribution in CDCL solvers yields additional motivation to improve on existing clause deletion schemes. These are not only needed to speed up unit propagation steps but are indispensable tools to avoid getting stuck in the tail of the distribution and ultimately avoid excessively long solving times. Additionally, the long-tailed property theoretically explains why completely flushing the learned clause database and forgetting the partial assignment (so-called resetting) is useful for CDCL algorithms.

We furthermore provided a hypothesis for the suitability of the Weibull distribution by invoking the Fisher–Tippett–Gnedenko theorem. It seems reasonable that runtimes are heavily influenced by the quality of the “best” and “worst” clauses. An analysis of these clause qualities, especially in the context of LBD [10], seems like a fruitful pursuit for further research.

A refined approach in future research could also investigate the influence of different batches of learned clauses on the runtime (e. g., are clauses learned later always more helpful than clauses learned earlier “towards the way of” a solution?). It might also be interesting to examine the quality of clauses from trimmed proofs with the approach shown in this paper. Since Glucose 4.1 has a feature to dynamically adapt its search strategy during a run, a very interesting follow-up investigation could also consist of classifying the clusters of extended instances by the search strategies used by the solver.

## 8 Generated data and evaluations

We have provided all data of this paper in the repository [43] (made available under 10.5281/zenodo.6642166). This collection contains the scripts for obtaining the sets L and reconstructing our sampled sets *L*. Furthermore, all data obtained by calling CDCLSolver(F∪L) can be found, where CDCLSolver ∈ {Glucose 4.1, Glucose 4.1 + ChanseokOh, MiniSAT}. Additionally, we included visual and statistical evaluations used in this paper.

## Supporting information

S1 TableInstance pool.This supporting table describes the instances used for our experiments with Glucose 4.1. The instance bivium-40–200-0s0–0x92fc13b11169afbb2ef11a684d9fe9a19e743cd6aa5ce23fb5–19 was abbreviated by bivium-40–200 in the table. The column *SAT/UNSAT* indicates whether the instance is satisfiable (SAT) or unsatisfiable (UNSAT). Note that extending an instance with a set L⊆L preserves the satisfiability of the instance. The column *cen* denotes the number of censored data points of each instance during the Glucose 4.1 trials, i. e., $\sum_{j=1}^{5000}{\text{cen}}_{j}$, where cen_*j*_ is the censoring indicator introduced in Eq (2). The columns *Z*_time_ and *p*_time_ report the results of the *t*-test for CPU time as described in Section 4. Similarly, the columns *Z*_confl_ and *p*_confl_ report the results of the *t*-test for the number of conflicts as described in Section 4. In both cases, a negative value of the test statistic *Z* signifies a positive effect for the mean of the extended instances. All values were rounded to two places. The values “—” in the table denote the two instances where complications in the *t*-test for the censored number of conflict data occurred. The table itself can be found on the following pages in landscape mode.(CSV)
